# Commentary: Sleep Changes without Medial Temporal Lobe or Brain Cortical Changes in Community-Dwelling Individuals with Subjective Cognitive Decline

**DOI:** 10.3389/fneur.2017.00262

**Published:** 2017-06-09

**Authors:** Claudio Liguori, Francesca Izzi, Nicola Biagio Mercuri, Fabio Placidi

**Affiliations:** ^1^Sleep Medicine Centre, Neurophysiopathology Unit, Department of Systems Medicine, University of Rome “Tor Vergata”, Rome, Italy; ^2^Fondazione Santa Lucia IRCCS, Rome, Italy

**Keywords:** obstructive sleep apnea, Alzheimer’s disease, beta amyloid, sleep, polysomnography, subjective cognitive decline

Over the last years, subjective cognitive decline (SCD) emerged as a possible preclinical Alzheimer’s disease (AD) condition. Accordingly, SCD patients showing pathological CSF beta-amyloid_42_ (Aβ_42_) concentrations are currently considered at risk of developing AD neurodegeneration (1). Since SCD may be identified as an early stage of AD pathology, therapeutic strategies performed in SCD patients may possibly stop the chain of events leading to AD neurodegeneration (2). To this end, early recognition of SCD is mandatory in order to possibly prevent and/or delay AD onset and progression through appropriate interventions (3–6).

It is well known that objective and subjective sleep impairment frequently affect AD patients, significantly concurring to cognitive decline (7, 8). Furthermore, sleep dysregulation may directly induce AD preclinical changes, such as a reduction of CSF Aβ_42_ levels and a greater cerebral Aβ_42_ deposit in AD-sensitive brain regions (4, 5, 9). Therefore, it has been suggested that sleep alteration promotes AD neurodegenerative changes through the glymphatic system malfunction, which during night-time sleep physiologically cleans the brain from neurotoxic products (i.e., Aβ_42_) accumulated during wakefulness (10).

Lauriola and co-authors recently published an important paper in *Alzheimer’s and Dementia Journal* suggesting that sleep changes may precede medial temporal lobe or brain cortical atrophy in patients affected by SCD (3). In particular, they documented, through actigraphs and sleep questionnaires, that objective sleep impairment, and not subjective sleep disturbances, is associated with SCD diagnosis (3). In fact, on the one hand, SCD patients showed lower sleep efficiency (SE) and higher wakefulness after sleep onset (WASO) compared to age-matched healthy controls (3). On the other hand, sleep complaints did not differ between SCD patients and controls. Moreover, SCD patients did not differ in brain magnetic resonance (MRI) measures compared to controls (3). Therefore, authors concluded that objective sleep impairment may be an early sign of neurodegeneration in SCD patients, preceding brain MRI pathological changes (3). Although the consistent impact of the findings proposed by Lauriola and colleagues on the current literature focused on sleep and neurodegeneration, some issues emerged. First, actigraph was used to objectively quantify sleep, while polysomnography (PSG) is actually considered the gold standard and the most recommended tool for assessing sleep quality and continuity in the adult population. Second, taking into account the wide use of CSF biomarkers in several clinical settings, the lack of CSF AD biomarkers analysis in the population of SCD patients studied by Lauriola and co-authors should be addressed. In fact, in the AD pathological process, brain MRI changes follow beta-amyloid plaques deposition after the reduction of CSF beta-amyloid_42_ (Aβ_42_) levels (11). Therefore, it is not surprising that SCD patients may not show significant brain MRI pathological changes, which usually occur later in the AD process. Finally, even if SCD is not associated with pathological scores at the neuropsychological testing, Lauriola and colleagues did not find differences in the complete neuropsychological assessment between SCD patients and controls. This result is in contrast with previous studies documenting memory deficits in SCD patients (12). Hence, considering that sleep changes frequently affect SCD patients (3), how could we detect this useful data in the clinical practice in order to improve screening and follow-up of SCD patients? Moreover, once reduced SE and increased WASO have been identified, how could we set preventive strategies against AD in SCD patients?

The reduction of SE and the increase of WASO are two markers of sleep quality reduction, which can be due to several sleep disturbances, including obstructive sleep apnea (OSA). OSA is actually considered the most frequent sleep disorder in both adults and elderly. Notably, it is often associated with several morbidities, such as cardiovascular and cerebrovascular diseases, diabetes, osteoporosis, cranial neuropathies, and cognitive impairment (13–15).

Our group has documented recently that OSA may induce early but possibly modifiable AD biomarkers changes in SCD patients (6). In particular, PSG, CSF biomarkers analysis (tau proteins and Aβ_42_), and neuropsychological testing were performed in a population of SCD patients in order to evaluate the impact of OSA on sleep, cognition, and CSF AD biomarkers. We demonstrated that OSA may significantly alter both CSF AD biomarkers and neuropsychological tests in SCD patients by reducing sleep quality and continuity and producing night-time intermittent hypoxia. Hence, we supposed that OSA could concur to preclinical AD pathological changes. Thus, we compared OSA group with a sample of OSA patients successfully treated by continuous positive airway pressure (CPAP), which is considered the gold standard treatment. We found that OSA patients on CPAP therapy did not show pathological changes in AD biomarkers; therefore, we hypothesized that CPAP may recover AD biomarkers changes and may revert the AD pathological cascade, by restoring sleep and nocturnal oxygen saturation.

In conclusion, we strongly recommend the screening of SCD patients for sleep disorders using objective assessments (i.e., PSG), since OSA may induce changes in AD biomarkers. Consistently, the early diagnosis of OSA and its treatment with CPAP can limit the AD pathological cascade, thus preserving the human brain from AD pathology.

## Author Contributions

CL: literature review and manuscript preparation. FP, FI, and NM: critical review of the manuscript for clarity and intellectual contents.

## Conflict of Interest Statement

The authors declare that the research was conducted in the absence of any commercial or financial relationships that could be construed as a potential conflict of interest.
